# Coronary atherosclerosis imaging by CT to improve clinical outcomes

**DOI:** 10.1016/j.jcct.2019.03.007

**Published:** 2019

**Authors:** Michelle C. Williams, David E. Newby, Edward D. Nicol

**Affiliations:** a University of Edinburgh/British Heart Foundation Centre for Cardiovascular Science, Edinburgh, UK; b Edinburgh Imaging Facility QMRI, University of Edinburgh, Edinburgh, UK; c Royal Brompton and Harefield NHS Foundation Trust Departments of Cardiology and Radiology, London, UK; d National Heart and Lung Institute, Faculty of Medicine, Imperial College, London, UK

## Abstract

Coronary artery disease remains an important cause of morbidity and mortality world-wide. Coronary Computed Tomography Angiography (CCTA) has excellent diagnostic accuracy and the identification and stratification of coronary artery disease is associated with improved prognosis in multiple studies. Recent randomized controlled trials have shown that in patients with stable coronary artery disease, CCTA is associated with improved diagnosis, changes in investigations, changes in medical treatment and appropriate selection for revascularization. Importantly this diagnostic approach reduces the long-term risk of fatal and non-fatal myocardial infarction. The identification of adverse plaques on CCTA is known to be associated with an increased risk of acute coronary syndrome, but does not appear to be predictive of long-term outcomes independent of coronary artery calcium burden. Future research will involve the assessment of outcomes after CCTA in patients with acute chest pain and asymptomatic patients. In addition, more advanced quantification of plaque subtypes, vascular inflammation and coronary flow dynamics may identify further patients at increased risk.

## 1 Introduction

Cardiovascular disease remains an important cause of morbidity and mortality world-wide, responsible for 17.9 million deaths per year.^1^ Both the presence and extent of coronary artery disease identified on coronary computed tomography angiography (CCTA) is associated with an adverse prognosis in multiple single center studies and several large multicenter registries. Recent research has shown that management guided by CCTA is associated with improved outcomes for patients with suspected coronary artery disease. The potential mechanisms for the improvement in outcomes in patients undergoing CCTA include more accurate diagnosis, appropriate use of medical therapy and appropriate selection for revascularization. In addition to the identification of coronary artery stenoses, CCTA can also identify the overall burden of atherosclerotic plaque and plaque characteristics, which may be associated with increased risk.

## 2 Detection and prognosis

**Fig. 1:**
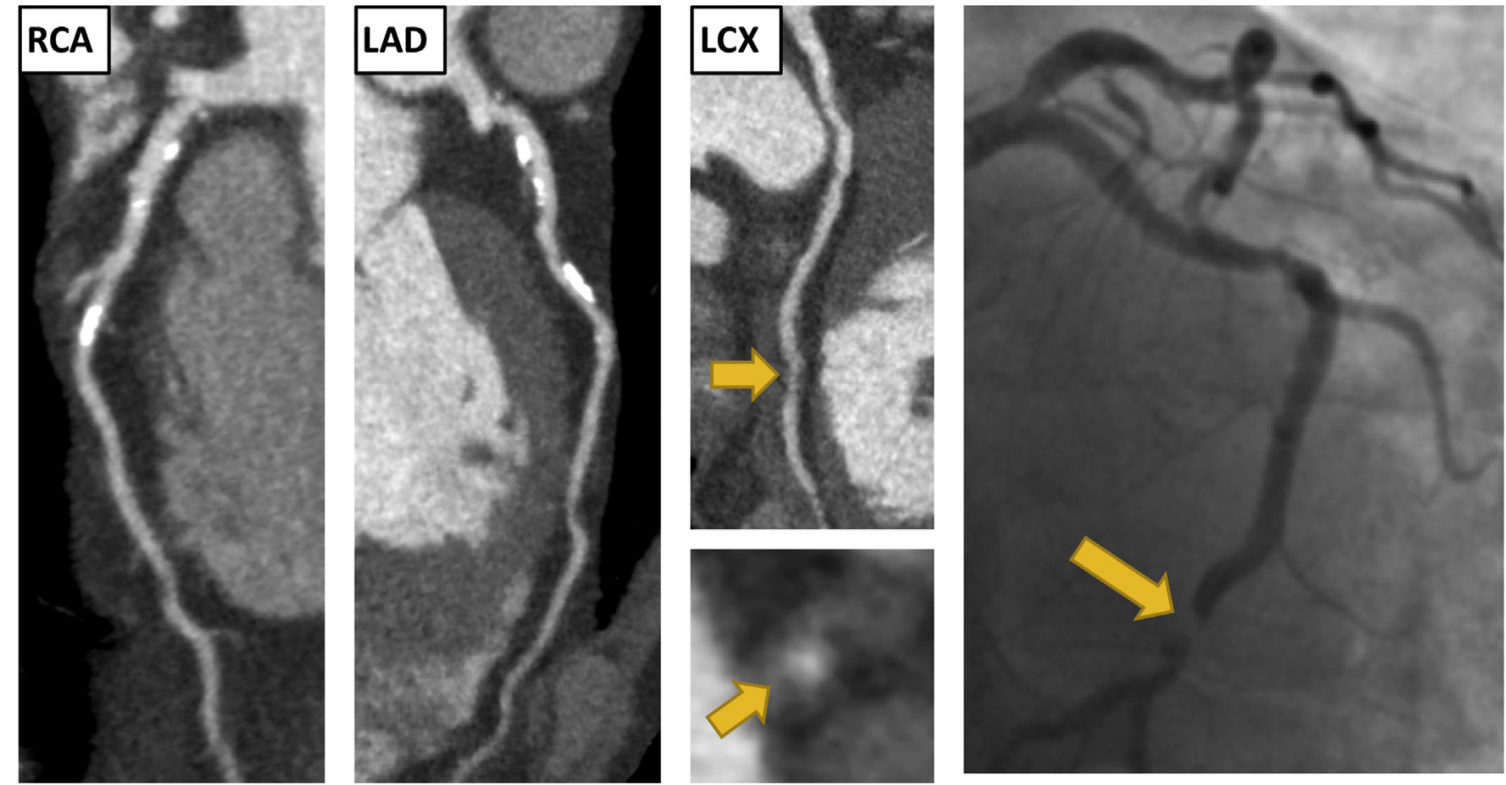
Clinical Vignette 1. A 65-year-old male presented to the rapid access chest pain clinic with symptoms of typical angina. He was a current smoker with a past medical history of gout. An exercise tolerance test was stopped early due to knee pain, but demonstrated no other abnormalities. His coronary artery calcium score was 283 Agatston units. He underwent a CCTA which showed moderate non-obstructive disease (CAD-RADS 3) He had mild calcified plaque in the left anterior descending (LAD) and right coronary artery (RCA), <50% stenosis. He had moderate non-calcified plaque with positive remodelling in the proximal and distal left circumflex artery (LCX), 50–70% stenosis. He presented 1 years later with an episode of acute chest pain on the background of 2 weeks of worsening chest pain. Electrocardiogram (ECG) on admission showed ST depression in the inferior leads. High sensitivity troponin I was elevated at 2060 ng/L. He was diagnosed with a non ST elevation myocardial infarction (NSTEMI). Invasive coronary angiography showed an occluded mid LCX (yellow arrows). There was also mild disease in the LAD and moderate disease in the small RCA. A bare metal stent was inserted into the LCX and he was asymptomatic on subsequent follow-up. . (For interpretation of the references to colour in this figure legend, the reader is referred to the Web version of this article.)

**Fig. 2:**
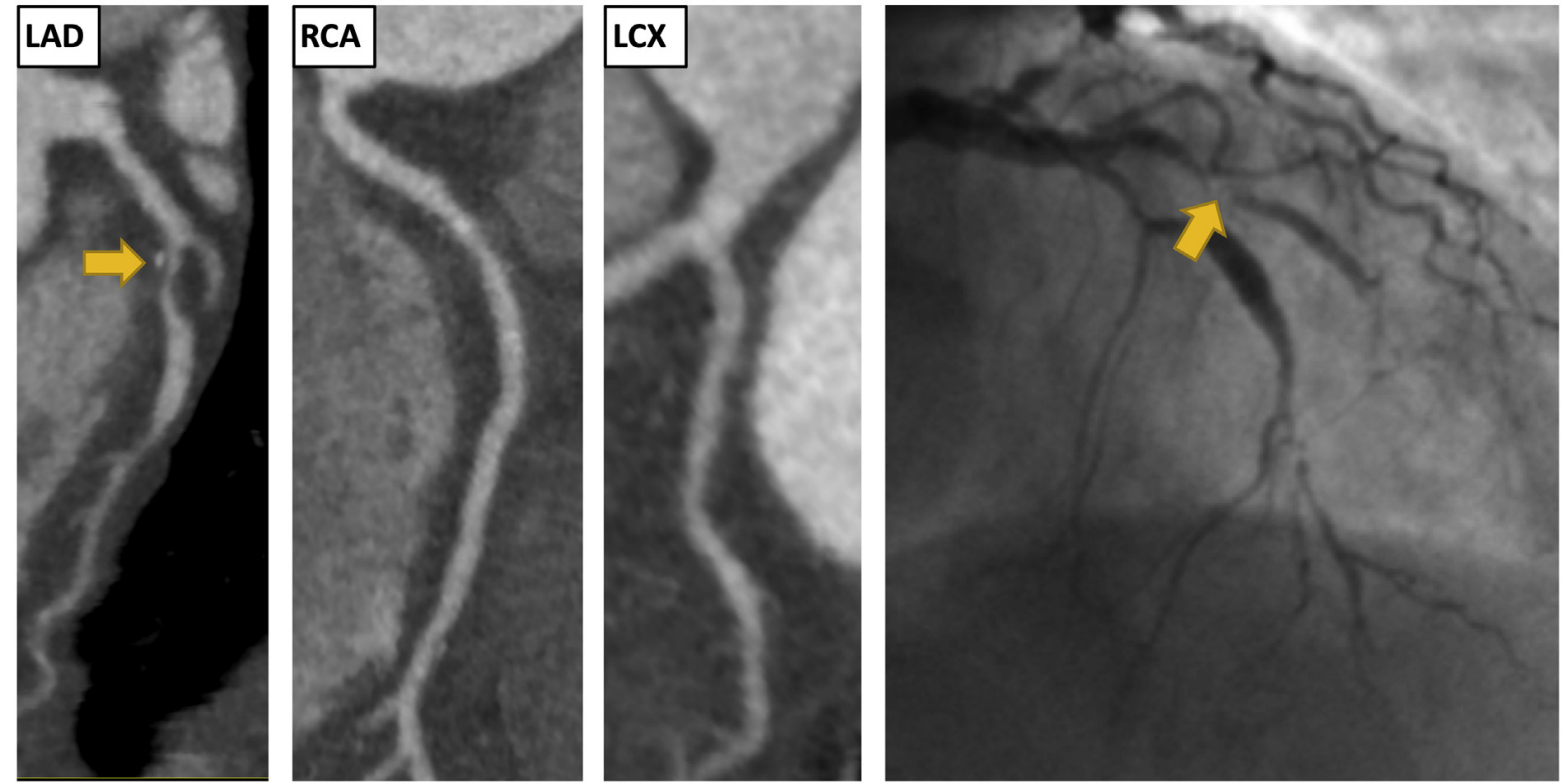
Clinical Vignette 2. A 67-year-old female presented to the rapid access chest pain clinic with atypical chest pain. She had hypertension but no other cardiovascular risk factors. Her estimated 10-year risk of cardiovascular disease was 14% (ASSIGN score). An exercise tolerance test was normal. Her coronary artery calcium score was 62 Agatston units. CCTA identified single vessel obstructive coronary artery disease (CAD-RADS 4A). There was severe mixed plaque in the proximal and mid LAD, >70% stenosis. The plaque in the proximal LAD demonstrated positive remodelling and spotty calcification. There was also mild disease in the mid RCA and proximal LCX, <50% stenosis. 4 years later she presented to the emergency department with acute chest pain. ECG showed ST depression in the inferior leads. High sensitivity troponin I was elevated at 387 ng/L. Invasive coronary angiogram showed an occluded LAD (yellow arrows) and mild disease in the LCX and RCA. The LAD was treated with a drug eluting stent. She had no further hospital admissions in the subsequent 3 years. . (For interpretation of the references to colour in this figure legend, the reader is referred to the Web version of this article.)

CCTA has an excellent diagnostic accuracy, both in terms of the ability to identify the presence of coronary artery plaque and the ability to identify obstructive coronary artery stenoses.^2^ The high negative predictive value means that CCTA can exclude the presence of coronary artery disease and help avoid any further unnecessary investigations or treatments. Conversely, CCTA can also risk stratify patients with coronary artery disease, including the appropriate selection of those who may benefit from revascularization (see Fig. 1, Fig. 2).^3^

On the background of excellent diagnostic accuracy, several single and multicenter registry studies have demonstrated that a normal CCTA is associated with an excellent prognosis.^4^, ^5^, ^6^ In contrast, the presence and magnitude of both non-obstructive and obstructive coronary artery disease is associated with subsequent prognosis.^6^, ^7^, ^8^, ^9^ This is true for both symptomatic and asymptomatic patients with a wide range of cardiovascular risk factors. The identification and quantification of coronary artery calcification (CAC), a surrogate marker of atherosclerosis, is also associated with a poor prognosis in a wide range of patient populations. Unlike CAC scoring, CCTA can identify plaque subtypes including both calcified and non-calcified plaque, and can identify the presence and severity of coronary artery stenoses. Several studies have shown that CCTA is additive to CAC in the assessment of prognosis.^10^

CCTA has excellent diagnostic accuracy for the detection of coronary artery disease and its ability to predict subsequent events was an important first stage in the adoption of this technology. However, over the past 5 years, several randomized controlled trials have addressed the important question of whether management based on CCTA can improve outcomes. These randomized controlled trials have set a benchmark for further research into diagnostic strategies for patients with suspected coronary artery disease.

## 3 Improved outcomes in patients with stable chest pain

Chest pain represents an important, and frequent, reason for consultation in both primary and secondary care.^11^^,^^12^ The many potential causes of chest pain mean that accurate diagnosis is an essential aspect of clinical care. Anginal chest pain due to coronary artery disease is an important diagnosis of both inclusion and exclusion for subsequent patient management. Recently two large multi-center randomized controlled trials and two smaller randomized trials, have addressed the question of prognostic outcomes after CCTA (Table 1). These include the Scottish Computed Tomography of the HEART trial (SCOT-HEART)^13^, ^14^, ^15^ and the Prospective Multicenter Imaging Study for Evaluation of Chest Pain (PROMISE) trial.^16^^,^^17^

**Table 1:** Randomized controlled trials of CCTA in patients with stable chest pain.^13^, ^14^, ^15^, ^16^, ^17^, ^18^, ^19^.

		PROMISE	SCOT-HEART	CAPP	Min et al.
Number of patients		10,003	4146	448	180
CCTA vs		Functional testing	Standard care	Stress ECG	MPI
Age (years)		61	57	59	56, 59
Female (%)		53	44	45	44
Pre-test probability (%)		53	47	45, 48	
Symptoms	Typical angina	12	35	34	32
	Atypical angina	78	24	8	23
	Non-anginal chest pain	11	41	67	27
Follow-up (months)		25	21, 58	12	2

PROMISE, Prospective Multicenter Imaging Study for Evaluation of Chest Pain; SCOT-HEART, Scottish Computed Tomography of the HEART; CAPP, Cardiac CT for the Assessment of Pain and Plaque; ECG, electrocardiogram; MIP, myocardial perfusion imaging; CCTA, computed tomography coronary angiography.

### 3.1 Study design of the multi-center randomized controlled trials

SCOT-HEART is a large multi-center trial based in the UK which assessed the use of CCTA in stable patients attending cardiology outpatient clinics.^13^ It recruited 4146 patients between 18 and 75 years, without excluding patients based on the presence of atrial fibrillation, body mass index or coronary artery calcium score. Patients were randomized to standard care or standard care plus computed tomography (CT). The primary outcome was the diagnosis of angina due to coronary artery disease at 6 weeks.^14^ Patients were followed-up for the primary clinical outcome of coronary heart disease death or non-fatal myocardial infarction at 5 years.^15^

PROMISE is a large multi-center trial based in the US which conducted a head-to-head comparison of CCTA with functional stress testing in just over 10,000 patients with suspected coronary artery disease.^16^ The functional testing arm included exercise electrocardiography (10%), exercise or pharmacologic nuclear stress testing (70%), and stress echocardiography (20%). The primary outcome was a composite of death, non-fatal myocardial infarction, hospitalization for unstable angina, or major procedural complication (including renal failure and anaphylaxis), and patients were follow-up for 2 years.^17^

### 3.2 Outcomes in the randomized controlled trials

The SCOT-HEART trial showed an improvement in outcomes in the CCTA group with a reduction in death from coronary heart disease or non-fatal myocardial infarction at both 2 (hazard ratio (HR) 0.62, 95% CI 0.38 to 1.01, p = 0.0527) and 5 years (HR 0.59, 95% confidence interval (CI), 0.41 to 0.84, p = 0.004).^12^^,^^13^ This was largely driven by reduction in the risk of non-fatal myocardial infarction. Thus, the SCOT-HEART study demonstrated for the first time that management based on the results of a non-invasive diagnostic test can improve coronary artery disease outcomes in a randomized controlled trial.

The PROMISE trial showed no differences in the primary composite endpoint of death, non-fatal myocardial infarction, hospitalization for unstable angina and major procedural complication between the CCTA and functional care groups (HR 1.04, 95% CI 0.83 to 1.29, p = 0.75) at 2 years.^17^ A statistical nuance is that these results failed to meet both superiority and non-inferiority criteria, leaving the impact of this study debated. However, consistent with the SCOT-HEART trial, there was a lower rate of death or non-fatal myocardial infarction in the CCTA group (HR 0.66, 95% CI 0.44 to 1.00, p = 0.049) at 12 months where they had 93% complete follow-up data.^17^ PROMISE suggests that management based on both strategies is safe and suitable for the assessment of patients with suspected coronary artery disease.

Two smaller randomized controlled trials have assessed outcomes in patients with stable chest pain. The CAPP (Cardiac CT for the Assessment of Pain and Plaque) trial was a single center randomized controlled trial comparing CCTA and exercise ECG which recruited 448 patients.^18^ It found no difference in major adverse cardiac events between groups, but this trial was underpowered for this outcome. A study by Min et al. randomized patients to CCTA or myocardial perfusion single-photon emission computed tomography and recruited 180 patients.^19^ At 55 days, no patient had a myocardial infarction or died, although this study was again underpowered. A meta-analysis has assessed the combined outcomes of these studies, including the 2 but not 5-year SCOTH-HEART results.^20^ It found that patients undergoing CCTA had a significant reduction in the annual rate of myocardial infarction (rate ratio 0.69, 95% CI 0.49 to 0.98, p = 0.038) but no difference in all-cause mortality (rate ratio 0.96, 95% CI 0.72 to 1.28, p = 0.78).^20^

## 4 Mechanisms for improved outcomes in patients undergoing CCTA

There are a variety of mechanisms by which patients undergoing CCTA may benefit from subsequent improved outcomes. This includes more accurate diagnosis, optimization of medical therapy, appropriate use of subsequent investigations including invasive coronary angiography and appropriate selection for revascularization.

### 4.1 CCTA improving diagnosis

For over 20 years, risk stratification models have been central to the diagnosis of patients with chest pain.^21^ However these models have several limitations. First, the data used to create them was obtained prior to the development of modern management strategies. Second, they include patients with a limited range of age, sex and ethnicity. Third, they led to a limited stratification of patients, with many in the pre-test probability range of 10–90%.^22^ Thus the diagnostic ability of risk prediction models is limited.

CCTA provides an accurate answer to the question of whether or not the patient has coronary artery disease and can also provide information as to the likelihood of whether the patient's symptoms are likely to be secondary to coronary artery disease. In the SCOT-HEART trial, CCTA led to a change in diagnosis in 23% of patients, compared to 1% in the standard care arm (p < 0.001).^14^ Improvements in the accuracy of the diagnosis are important in providing the optimal clinical care. In addition to diagnostic accuracy, CCTA can also improve the certainty of a diagnosis. In SCOT-HEART, CCTA improved the certainty of the diagnosis of coronary artery disease (relative risk (RR) 2.56, 95% CI 2.33 to 02.79, p < 0.0001) and angina due to coronary artery disease (RR 1.79, 95% CI 1.62 to 1.96, p < 0.001). The certainty of a diagnosis has important implications for the subsequent actions of both physicians and patients.^23^ It should be noted that exclusion of CAD, whether in its entirety, or as a likely cause of symptoms, is as important as the confirmation of significant disease. This also informs the most appropriate management strategy, which may not include pharmacological or physical intervention.

### 4.2 CCTA and optimization of medical management

The use of CCTA can lead to changes in the medical management of patients. This includes the starting of new medications in patients who have coronary artery disease, or the stopping of unnecessary medications in patients who have been shown to have normal coronary arteries.

In the SCOT-HEART study, there was a change in medical treatments in 27% of patients in the CCTA group and 5% in the standard care group.^14^ This included changes in recommendations for both preventative (18% vs 4%, p < 0.001) and anti-anginal treatments (9% vs 1%, p < 0.001).^14^ A subsequent Danish registry study of 86,705 patients who underwent functional testing or CCTA also showed that the use of CCTA was associated with changes in medical management.^24^ They found that there was increased use of preventative medications after CCTA compared to functional testing, including statins (26% vs 9%, p < 0.001) and aspirin (13% vs 9%, p < 0.001).^24^ Several other smaller studies have shown that CCTA leads to the intensification of preventative medical therapies^19^^,^^25^^,^^26^ and subsequent improvements in cholesterol measurements.^27^

Another important factor that CCTA can influence is the continuation of long-term preventative medications. A variety of patient and clinician factors can impact compliance with medication recommendations, including demographic, clinical, socio-economic, and belief-related factors.^28^^,^^29^ The identification of CAC is associated with increased adherence to statin therapy, and patients with higher CAC score are more likely to adhere to statin therapy.^30^, ^31^, ^32^ In the SCOT-HEART trial, there was increased use of preventative therapy in patients in the CCTA group throughout follow-up, particularly in those with coronary artery disease.

Therefore, it can be surmised that changes in medical management after CCTA are likely to be important factors in the improvement of outcomes after CCTA through both the individualization of medical treatment recommendations and improvement in subsequent adherence.

### 4.3 CCTA and appropriate use of downstream investigations

Initial criticism of CCTA was that it would merely increase the number of subsequent diagnostic tests that are performed. However, this has not been confirmed in long-term follow-up studies. The identification of coronary artery disease *per se* does not necessitate invasive imaging. Indeed, the majority of patients who undergo CCTA do not require any further imaging investigations. Invasive coronary angiography should be reserved for patients who are likely to require revascularization. In this respect, CCTA can be used as a “gate-keeper” for subsequent invasive investigation.

CCTA led to changes in investigations in 15% of patients, compared to 1% in the standard care group (p < 0.0001).^14^ This included the cancellation of investigations, particularly additional functional imaging, and the organisation of new investigations, primarily invasive coronary angiography. Although there was an early increase in the use of invasive coronary angiography in both the PROMISE and SCOT-HEART trials, the overall rates of invasive coronary angiography were similar in the CCTA group and the standard care group by 5 years of follow-up (HR 1.00, 95% CI 0.88, 1.13).^15^ This suggests the more appropriate targeted use of invasive coronary angiography after CCTA that reduced the need for subsequent downstream invasive angiography. The study by Min et al. also identified similar rates of invasive coronary angiography after CCTA and functional testing.^19^ A meta-analysis of CCTA studies showed that there was a trend towards more invasive coronary angiography after CCTA (OR 1.33, 95% CI 0.95 to 1.84, p = 0.09),^20^ but this did not include studies with long-term follow-up.

The rates of normal coronary arteries identified on diagnostic invasive coronary angiography varies depending on the center, indication or population, but has been quoted as being over 50%.^33^ Due to the potential for rare but serious complications, its invasive nature, and the cost, it is important to ensure the appropriate use of invasive coronary angiography. Indeed, in the era of non-invasive imaging, finding normal coronary arteries on invasive coronary angiography should be an uncommon event. The PROMISE trial showed that there were fewer invasive coronary angiograms showing normal coronary arteries in the CCTA group compared to the functional testing group (3.4% vs 4.3%, p = 0.02).^17^ Similarly in the SCOT-HEART trial, CCTA was associated with a decreased rate of normal coronary arteries (HR 0.39, 95% CI 0.23 to 0.68, p < 0.001).^34^ Other studies have also shown a reduction in the frequency of normal coronary arteries at invasive coronary angiography after CCTA, confirming that CCTA can be used as a gate-keeper for the use of invasive coronary angiography.^3^

### 4.4 CCTA and appropriate use of revascularization

CCTA can assist the appropriate selection of patients who may benefit from revascularization. In the PROMISE trial, CCTA was associated with an increase in the number of patients undergoing revascularization (6.2% vs 3.2%, p < 0.001).^17^ A meta-analysis of randomized controlled trials, which included the 2 but not 5-year follow-up of the SCOT-HEART trial, found that there was a small increase in revascularization after CCTA compared to usual care (7.9% vs 5.1%, p < 0.001). The Danish registry study also showed an increase in invasive coronary angiography and revascularization in the CCTA group after 120 days of follow-up.^24^ In the first year of follow-up in the SCOT-HEART trial, there was an increase in the use of revascularization in the CCTA group (HR 1.21, 95% CI 1.01 to 1.46, p = 0.042). However, at 5 years, there was no difference in the rate of revascularization in the CCTA or standard care groups (HR 1.07, 95% CI 0.91 to 1.27), because beyond one year, there were higher rates of coronary revascularization in the standard care group (HR 0.59, 95% CI 0.38 to 0.90, p = 0.015). This suggests that the CCTA can guide the early selection of appropriate patients for both invasive coronary angiography and revascularization. It also suggests that early and more targeted treatment in the CTCA group prevented downstream longer-term disease progression, whereas there was declaration of unrecognized disease in the standard care group requiring further investigation and intervention.

## 5 CCTA and outcomes in patients with acute chest pain

Previous research into the use of CCTA in the Emergency Department has primarily focused on improving the time-to-diagnosis. Several randomized controlled trials have been performed in patients with a low to intermediate pre-test probability (Table 2). These have established that the presence of normal coronary arteries in patients presenting with acute chest pain is associated with a good prognosis and low risk of subsequent cardiac events. They have also shown that CCTA is associated with reduced time to-diagnosis, reduced length of hospital stay and reduced cost.^35^^,^^36^ However, a meta-analysis of the randomized controlled trials in patients with acute chest pain found that there was no difference in the outcomes of all-cause mortality, myocardial infarction or major adverse cardiac events between the CCTA and standard care groups.^37^

**Table 2:** Randomized controlled trials of CCTA improving outcomes in acute chest pain^35^^,^^36^^,^^66^^,^^67^.

	ACRIN-PA	BEACON	CATCH	CT-COMPARE	CT-STAT	Nabi et al.	PERFECT	PROSPECT	ROMICAT-II
Number of patients	1368	490	576	562	699	598	395	400	1000
CCTA vs	Stress ECG, stress imaging	Stress ECG, MPI	Stress ECG, MPI	Stress ECG	MPI	MPI	Stress echo., MPI	MPI	Stress ECG, Stress echo., MPI
Age (mean years)	50	54	56	52	50	53	60	57	54
Female (%)	54	47	45	42	54	56	54	63	47
Follow-up (months)	12	1	19	12	6	7	12	12	1

ACRIN-PA, CT Angiography for Safe Discharge of Patients with Possible Acute Coronary Syndromes; BEACON, Better Evaluation of Acute Chest Pain with Coronary Computed Tomography Angiography; CATCH, CArdiac cT in the treatment of acute CHest pain; CT-COMPARE, CT Coronary Angiography Compared to Exercise ECG; CT-STAT, Coronary Computed Tomographic Angiography for Systematic Triage of Acute Chest Pain Patients to Treatment; PERFECT, Prospective First Evaluation in Chest Pain; PROSPECT, Prospective Randomized Outcome trial comparing radionuclide Stress myocardial Perfusion imaging and ECG-gated coronary CT angiography; ROMICAT-II, Rule Out MI/ischaemia Using Computer Assisted Tomography; ECG, electrocardiogram; MPI, myocardial perfusion imaging; echo., echocardiography.

Most of the previous studies of CCTA in the emergency department occurred prior to the adoption of high sensitivity troponin. One small trial, BEACON (Better Evaluation of Acute Chest Pain with Coronary Computed Tomography Angiography), employed measurement of high sensitivity troponin in all patients. This small study did not show any difference in outcomes between the groups. Post-hoc analysis of high sensitivity troponin in the ROMICAT II study identified a group of patients who could be discharged with good outcomes based on high sensitivity troponin and risk factors alone.^38^ Further randomized controlled trials are underway to assess the outcomes of patients undergoing CCTA in the Emergency Department including the RAPID-CTCA (Rapid Assessment of Potential Ischaemic Heart Disease with CTCA; NCT02284191) and TARGET-CTCA (Troponin in Acute chest pain to Risk stratify and Guide EffecTive use of Computed Tomography Coronary Angiography) trials.

## 6 CCTA and asymptomatic patients

In asymptomatic patients, the identification of CAC is associated with clinical outcomes and a zero CAC score associated with an excellent prognosis.^39^^,^^40^ In addition, the identification and stratification of coronary artery disease on CCTA predicts prognosis in registry studies of asymptomatic patients.^41^ The identification of coronary artery disease in asymptomatic patients therefore moves us away from primary prevention, and towards a new form of secondary prevention of coronary artery disease.

To date only one randomized controlled trial has assessed the use of CCTA in asymptomatic patients. The FACTOR-64 trial (Screening For Asymptomatic Obstructive Coronary Artery Disease Among High-Risk Diabetic Patients Using CT Angiography, Following Core 64) recruited 900 patients with type 1 or 2 diabetes without a previous history or symptoms of coronary artery disease.^42^ Patients were randomized to either undergo CCTA or standard care. After 4 years, there was no difference in the primary end point of all-cause mortality, non-fatal myocardial infarction or unstable angina requiring hospitalization (HR 0.80, 95% CI 0.49 to 1.32, p = 0.38).^42^ However, this was a small trial, and a large proportion of patients were already taking preventative medication prior to CCTA. Therefore, the role of CCTA in the assessment of asymptomatic patients remains uncertain. Other non-randomized prospective studies support the utility of CCTA to identify coronary artery disease in asymptomatic patients.^43^ Future research will assess the role of CCTA in the broad population of patients with suspected coronary artery disease, including the SCOT-HEART 2 trial.

## 7 Impact of adverse plaque characteristics on outcomes

Although the presence of coronary artery stenosis is associated with subsequent events, most myocardial infarctions occur in segments with non-obstructive rather than obstructive disease.^44^^,^^45^ Thus, the identification other plaque characteristics which are associated with prognosis is important. An important advantage of CCTA is that in addition to the identification of coronary artery stenosis, it can also assess plaque characteristics. This can be in the form of visual assessment, or more recently using software capable of semi-automated quantitative assessment.

Simple assessment of plaque involves the identification of calcified, non-calcified and mixed subtypes. The stratification of plaques with this method has shown that non-calcified plaques in particular are associated with acute coronary events.^46^ More detailed visual assessment includes the identification of “adverse plaque features” including positive remodelling, low attenuation plaque, spotty calcification and the “napkin-ring” sign. These features are associated with markers of histological vulnerability^47^ and with prognosis in several studies.^48^, ^49^, ^50^, ^51^, ^52^, ^53^ A meta-analysis found that the risk of future ACS was increased in patients with high-risk plaque (OR 12.1, 95% CI 5.24 to 28.1, p = 0.0001).^54^

In a study of 3158 patients followed up for 4 years, Motoyama et al. found that the presence of positive remodelling or low attenuation plaque was associated an increased likelihood ACS.^50^ Adverse plaques are frequent on CCTA performed for both stable and acute chest pain occurring in 15% of patients in the PROMISE trial, 35% in ROMICAT II and 34% in SCOTHEART.^54^, ^55^, ^56^ In ROMICAT II, adverse plaque (positive remodelling, low attenuation plaque, spotty calcification or “napkin-ring”) were more frequent in patients with ACS (odds ratio 8.9, 95% CI 1.8 to 43.3, p = 0.006).^55^ In the PROMISE trial, adverse plaques (positive remodelling, low attenuation plaque, or “napkin-ring”) were associated with increased risk of death, myocardial infarction or hospitalization for unstable angina at 2 years (HR 2.73, 95% CI 1.89, 3.93).^56^ In the SCOT-HEART trial, adverse plaques (positive remodelling and/or low attenuation plaque) were associated with an increased risk of coronary heart disease death or myocardial infarction (HR 3.01, 95% CI 1.61 to 5.63, p = 0.001), but this was not independent of CAC.^57^ This suggest that the primary factor for long term prognosis is the overall plaque burden rather than the presence of an adverse plaque on non-invasive imaging. This is not surprising as the burden of coronary artery disease measured as coronary artery calcium score, number of vessels involved, or more complex scores are all predictive of outcomes. In addition, the development of atherosclerotic plaque is a continuous dynamic process. The fact that adverse plaques appear to be predictive of ACS and early outcomes, but not of late outcomes, is consistent with the theory of continuous plaque remodelling. An individual HRP may undergo subsequent stabilisation or subclinical rupture, rather than present with ACS. Therefore, to predict long term events, the presence of any plaque is more important than the type of plaque.

The retrospective nature of some studies to date, the high frequency of adverse plaque, and the low frequency of clinical outcomes, means that the relevance of the assessment of adverse plaque in clinical practice is uncertain. In addition, the visual assessment of atherosclerotic plaque is time consuming and associated with observer variability.^58^ Current research into the quantitative assessment of atherosclerotic plaque aims to address these issues. Quantitative plaque analysis has been used to assess plaque features associated with severe stenoses,^59^ myocardial perfusion defects^60^ and abnormal computed tomography fractional flow reserve (CT FFR).^61^ It has also been used to assess differences in plaque types between diabetic and non-diabetic patients,^62^ and assess plaque progression.^62^ The prognostic implication of quantitative plaque assessment has been assessed in registry and case control studies.^63^^,^^64^ In a study of 2748 patients, the quantification of total, non-calcified and low-density plaque volumes and contrast density drop were predictive of cardiac death at 5 years of follow-up.^64^ Future research will improve the automation of these techniques but there is a need to assess their impact in randomized studies of outcomes. In addition, advanced computational techniques seek to identify additional features of adverse plaques which are visible to the computer rather than the human eye.^65^ This may be complementary to other techniques which are under investigation such as CT assessment of vascular inflammation or vascular flow dynamics.

## 8 Conclusion

CCTA can improve outcomes by identifying patients who may benefit from medical therapy or revascularization. It can also identify patients who do not have coronary artery disease and avoid unnecessary investigation or life-long medication in these patients. Thus, by improving the accuracy of diagnosis and facilitating appropriate management, a diagnostic strategy including CCTA can improve outcomes for patients with suspected coronary artery disease. There is robust evidence for this in patients with stable chest pain and further research is underway in patients with acute chest pain and asymptomatic patients. The visual or quantitative assessment of plaque may further improve outcomes for patients with coronary artery disease in the future.

## Conflicts of interest

MCW has performed consultancy for GE Healthcare.
